# Linking shelter conditions to health: A multisystem analysis of stress, metabolism, and fecal microbiota in dogs

**DOI:** 10.1371/journal.pone.0350401

**Published:** 2026-07-01

**Authors:** Adul Saengthong, Janine L. Brown, Patcharapa Towiboon, Khanittha Punturee, Songphon Buddhasiri, Korakot Nganvongpanit, Veerasak Punyapornwithaya, Wuithipong Tocharoennirattisai, Jaruwan Khonmee

**Affiliations:** 1 Faculty of Veterinary Medicine, Chiang Mai University, Chiang Mai, Thailand; 2 Smithsonian National Zoo & Conservation Biology Institute, Front Royal, Virginia, United States of America; 3 Center of Elephant and Wildlife Health, Chiang Mai University Animal Hospital, Chiang Mai, Thailand; 4 Cancer Research Unit of Associated Medical Sciences (AMS-CRU), Faculty of Associated Medical Sciences, Chiang Mai University, Chiang Mai, Thailand; Chattogram Veterinary and Animal Sciences University, BANGLADESH

## Abstract

Variations in space allocation and husbandry practices can significantly affect the health and welfare of long-term shelter dogs. This study compared adrenal, metabolic, and microbiological health indicators among dogs from three shelters in Thailand: Shelter A – low-density (20.3 m²/dog), adequate enclosure size (101.9 m²), and an enrichment program; Shelter B – medium-density (3.9 m²), large enclosures (150 m²); and Shelter C – high density (3.0 m²), one communal space (800 m²), and no enrichment. Fecal glucocorticoid metabolite (fGCM) concentrations, oxidative stress (malondialdehyde, MDA), metabolic parameters (glucose, insulin, fructosamine, lipid profiles), and fecal microbiota composition and diversity were measured and analyzed in relation to management factors. Dogs in Shelter A exhibited the lowest fGCM concentrations (207.41 ± 4.55 ng/g), normal metabolic profiles, and richer, more even fecal microbiota. In contrast, dogs in Shelter C, a temple-based facility with more crowding and limited space, exhibited higher fGCM (278.71 ± 10.44 ng/g) and fructosamine concentrations, reduced alpha diversity, and a highly skewed Firmicutes-to-Bacteroidota ratio, suggesting possible adrenal cortical and microbial dysbiosis. Shelter B presented intermediate values across most parameters and management inputs. These findings show that management practices, including space allocation and enrichment, can influence stress physiology, metabolic health, and fecal microbiota composition in long-term sheltered dogs.

## 1. Introduction

Global overpopulation of stray dogs and cats poses a serious threat to public health, animal welfare, and environmental sustainability [1]. Stray animals often suffer from disease, malnutrition, and injury, while contributing to zoonotic disease transmission, traffic hazards, and conflict with humans [2,3]. Unchecked breeding, pet abandonment, limited access to sterilization services, and low public awareness, especially in rapidly urbanizing areas with inadequate animal management infrastructure, are among the contributing causes [4]. In Southeast Asia, and particularly Thailand, stray dog overpopulation is widespread [5]. To control the problem, local Thai governments and non-governmental organizations (NGOs) have established long-term shelters, sterilization campaigns, and rabies vaccination programs [6]. While shelters contribute to public safety and help regulate stray populations, concerns remain regarding their effects on animal welfare. Dog behavior and physiological well-being are influenced by shelter conditions, particularly by enclosure size, enrichment, and types of human interactions [7–9]. For example, space allocation per dog has been linked to stress and metabolic parameters, and gut microbiota composition [10,11]. Although awareness of animal welfare is increasing across the region, empirical research examining the effect of shelter management practices on dog well-being in Asia remains limited.

Welfare assessment of shelter animals typically involves behavioral observations and measures of physiological indicators [12]. Common behavioral signs of stress in dogs include stereotypies, fearfulness, and aggression [13]. Physiologically, fecal glucocorticoid metabolites (fGCMs) offer a reliable, non-invasive measure of hypothalamic-pituitary-adrenal (HPA) axis activity and are increasingly used to assess chronic stress in a variety of species [14]. In dogs, elevated fGCM concentrations have been linked to poor housing conditions, high social density, and unpredictable environments [15,16]. However, hormonal indicators alone may not fully capture subclinical changes in physiological or health status. Therefore, incorporating additional biomarkers such as metabolic parameters and gut microbiota profiles can provide a more comprehensive understanding of welfare in shelter settings [12,17].

Metabolic markers such as malondialdehyde (MDA), fructosamine, insulin, glucose, triglycerides, cholesterol, low-density lipoprotein (LDL), and high-density lipoprotein (HDL) reflect the physiological impacts of chronic stress, including oxidative stress, energy imbalance, and impaired glucose or lipid metabolism [18–20]. MDA, a byproduct of lipid peroxidation, is widely recognized as an oxidative stress marker, and its elevation has been linked to increased physiological stress and inflammatory responses in dogs [21,22]. Similarly, altered levels of insulin, glucose, triglycerides, and cholesterol may signal shifts in energy metabolism due to environmental stressors or dietary inconsistencies [23,24]. Consequently, in shelter environments, particularly those characterized by overcrowding or suboptimal conditions, dogs may experience long-term metabolic dysregulation [25].

Chronic stress can also impair gut microbial balance, another emerging indicator of animal health and welfare [17]. The gut microbiome plays a central role in modulating immune function, nutrient metabolism, and even behavior through the gut-brain axis [26,27]. In dogs, shifts in microbial composition have been associated with dietary changes, stress, illness, and environmental conditions [28–30]; therefore, stress-related microbiota shifts may affect host homeostasis, especially in confined shelter populations. Thus, analyzing gut microbial composition in conjunction with stress and metabolic markers may reveal how differing management practices impact canine welfare by integrating indicators across hormonal, metabolic, and microbiological levels.

The amount of space allocated per animal in shelters is an important yet inconsistently applied welfare parameter. Crowding may lead to increased aggression, resource competition, and behavioral stress, while more generous space provisions may allow for rest, retreat, and reduced social pressure [10,31]. However, due to economic and infrastructural limitations, many shelters operate under space constraints, resulting in diverse management models and varying degrees of compliance with animal welfare standards [32,33]. Despite growing recognition of these issues, little is known about how differences in spatial management simultaneously affect physiological stress, metabolic balance, and gut microbiota composition in long-term sheltered dogs. Addressing this gap is crucial for developing evidence-based strategies to improve welfare and management practices, particularly in under-researched regions such as Asia.

The present study aimed to evaluate the welfare status of dogs housed in three shelters in Chiang Mai, Thailand, each characterized by different area-per-dog ratios and management strategies (see Saengthong et al., 2025). Using an integrative approach, we measured adrenal (fGCM), oxidative stress (MDA), and metabolic (fructosamine, glucose, insulin, triglycerides, cholesterol, LDL, and HDL) markers, as well as fecal microbiota profiles in dogs from each shelter. We hypothesized that dogs in shelters with larger area/dog ratios would exhibit lower stress levels, more normal metabolic profiles, and a healthier fecal microbiota composition compared to those housed in more crowded or resource-limited environments.

This multifactorial welfare assessment aims to provide empirical evidence linking shelter design and management to physiological and microbial outcomes in dogs. The results will offer insights for shelter practitioners, policymakers, and animal welfare advocates by identifying quantifiable welfare indicators that are affected by spatial and environmental factors. Ultimately, this study will contribute to the growing body of literature advocating for science-based shelter management practices that prioritize both physical health and psychological well-being of companion animals.

## 2. Methods

### Ethical consent

This study was approved by the Faculty of Veterinary Medicine, Chiang Mai University (CMU) Research Ethics Committee (Approval No. R22/2566). All methods were carried out in accordance with relevant guidelines and regulations. The reporting of this study adheres to the ARRIVE guidelines.

### Animals and sample collection

Three dog shelters in Chiang Mai Province, Thailand, evaluated previously as follows; Mae Taeng Shelter (Shelter A), Doi Saket Shelter (Shelter B), and Muang Shelter (Shelter C) [31], participated in this study. Shelters A, B, and C housed 511, 538, and 266 dogs, respectively, although only a subset of dogs at each shelter were evaluated for the study. All dogs included in this study were neutered, mixed-breed, and reported by shelter staff as healthy (Table 1). Each participating shelter confirmed that dogs had undergone veterinary health checks prior to sampling. Shelter A consisted of 102 pens with an average area of 101.9 m², Shelter B had 14 pens with an average area of 150 m², and Shelter C housed all dogs in a single communal area with a total area of 800 m². The average living space per dog in Shelters A, B, and C was 20.3 m², 3.90 m², and 3.0 m², respectively.

**Table 1:** Summary of dogs included in the study across three shelters in Chiang Mai, Thailand.

Characteristic	Shelter A (n = 511)	Shelter B (n = 538)	Shelter C (n = 266)
**Breed**	Mixed breed	Mixed breed	Mixed breed
**Age range (years)**	1.5–7	1.5–7	1.5–7
**Sex ratio (M:F)**	1.00: 1.18 all neutered	1.00: 1.23 all neutered	1.00: 1.24 all neutered
**Duration in shelter (year)**	4.06 ± 0.08 (1–8)	3.49 ± 0.07 (1–8)	3.28 ± 0.09 (1–7)

As described previously [31], Shelters A and B conducted routine pen cleaning twice daily, once in the morning and once in the evening (0800 and 1700 hours). In contrast, Shelter C performed cleaning only once per day, in the morning. Similarly, Shelters A and B provided food to the dogs twice daily during the same time periods, whereas Shelter C provided food once daily in the afternoon, between approximately 1500 and 1600 hours. All three shelters provided commercial dry dog food commonly available in Thailand, sourced through both shelter purchases and public donations, resulting in varied and inconsistent feeding regimens. Shelter A also offered dry or canned food and occasionally supplemented meals with cooked meat (e.g., boiled chicken). Shelter B sometimes substituted dry food with home-cooked meals, primarily rice mixed with small amounts of vegetables or meat. Shelter C, operating with limited funding, provided some commercial dry food but primarily relied on boiled rice with minimal or no animal protein. There were notable differences in both on-site veterinary services and environmental enrichment across Shelters. Shelter A had an on-site veterinary clinic and enrichment provisions, whereas Shelter C lacked both. Additionally, Shelter A allowed volunteers and visitors to interact with the animals through activities such as dog walking or playtime in designated areas. Enrichment was provided through toys placed in the pens and a swimming pool that was available for dogs to play and relax.

A total of 900 fresh fecal samples (n = 300 per shelter) were collected between 0600 and 0800 hours over a 1-month period in April and May. To minimize environmental contamination, all samples were collected immediately after defecation using sterile disposable gloves and sterile plastic zip-lock bags. Samples were placed into a sterile plastic zip-lock bag and frozen at −20°C within 1 hour of collection. Of those, a subset of 20 samples per shelter was used for fecal microbiome analysis.

Blood samples (n = 60 per shelter), 5 ml from either the cephalic (forelimb) or saphenous (hindlimb) vein, were obtained between 1300 and 1530 hours for the analysis of metabolic markers. Blood was centrifuged at 1,500 x g for 10 minutes, and the serum frozen and stored at −20°C.

### Fecal extraction and fGCM analysis

All chemicals were sourced from Sigma Chemical Company (St. Louis, MO, USA), unless specified otherwise. The extraction method was modified from Brown et al. [34]. Wet fecal samples were dried in a standard oven at 60°C for 24–48 hours, and subsequently stored at −20°C until hormone extraction. Before extraction, dried fecal samples were thawed at room temperature (RT), thoroughly homogenized, and 0.2 g (±0.01 g) of powdered feces placed into a glass tube containing 90% ethanol (v/v) in distilled water. Each sample underwent two extractions by boiling in a 96°C water bath for 20 minutes, with additional ethanol added as needed to keep from boiling dry. Following extraction, samples were centrifuged at 1,200 × g for 20 minutes. The resultant supernatants were combined and evaporated to dryness in a 50°C water bath. Dried extracts were reconstituted in 3 mL of ethanol by vortexing for 1 minute, then redried and redissolved in 50% methanol by vortexing before analysis. Final extracts were preserved at −20°C until fGCM analysis.

fGCM concentrations were quantified using a double-antibody enzyme immunoassay (EIA) employing a polyclonal rabbit anti-corticosterone antibody (CJM006, Coralie Munro, UC Davis, CA, USA). Each 96-well microtiter plate was pre-coated with 150 μL of anti-rabbit IgG (0.01 mg/mL) incubated at RT for 15−24 hours. Following incubation, wells were emptied and 250 μL of blocking solution was added and incubated at RT for another 15−24 hours. Wells were emptied and plates were dried at RT (Sanpla Dry Keeper, Sanplatec Corp., Auto A-3, Japan) using loose desiccant until humidity fell below 20%, then sealed in foil bags with a 1 g desiccant packet and stored at 4°C until required. For the EIA, 50 μL of sample extracts and corticosterone standards were added in duplicate to each well, followed by 25 μL of horseradish peroxidase (HRP)-conjugated corticosterone (diluted 1:30,000) and 25 μL of anti-corticosterone antibody (diluted 1:100,000). Plates were incubated at RT for 2 hours, followed by five washes with wash buffer. Subsequently, 100 μL of tetramethylbenzidine (TMB) substrate solution was introduced to each well and incubated at RT for 15–20 minutes. The enzyme reaction was terminated by the addition of 2 M sulfuric acid (H₂SO₄), and absorbance was quantified at 450 nm utilizing a microplate reader (TECAN, Männedorf, Switzerland). The assay sensitivity, based on 90% binding, was 0.14 ng/mL. All samples were analyzed in duplicate. Serial dilutions of dog fecal extracts were parallel to the corticosterone standard curve (y = 0.969x − 11.04, R² = 0.990), and recovery of added corticosterone to feces before analysis exceeded 90%. The intra-assay and inter-assay coefficients of variation (CVs) were <10% and <9.4%, respectively.

### Metabolic biomarker analyses

Serum MDA concentrations were measured using a thiobarbituric acid reactive substances (TBARS) method as described by Satitmanwiwat et al. [35]. In summary, 50 µL of serum and standard were combined with 750 µL of 0.44 M phosphoric acid, 250 µL of 42 mM thiobarbituric acid (TBA), and 450 µL of distilled water. The mixtures were boiled for 15 minutes, then cooled on ice for 5 minutes, and centrifuged at 1,500 rpm for 5 minutes. The supernatant was collected and measured at an absorbance of 532 nm using a UV-VIS spectrophotometer (Shimadzu, Japan). The quantities of MDA in samples were determined using a standard curve of MDA equivalents produced by the acid-catalyzed hydrolysis of 1,1,3,3-tetramethoxypropane (TMP) (5–80 µM).

Per the manufacturer’s guidelines, serum insulin concentrations were quantified with a Mercodia Bovine Insulin ELISA kit (Mercodia AB, Uppsala, Sweden). All reagents and samples were brought to RT prior to use. The enzyme conjugate (1X solution) and wash buffer (1X solution) were produced through dilution as directed. Calibrators, controls, and samples (25 µL each) were dispensed in duplicate into microplate wells, followed by the addition of 100 µL of enzyme conjugate solution. The plate was incubated on a shaker at 700–900 rpm for 2 hours at RT. The wells were subsequently washed six times with 350 µL of wash buffer. Subsequently, 200 µL of TMB substrate was introduced to each well, and the plate was incubated at RT for 15 minutes. The reaction was terminated by the addition of 50 µL of stop solution (0.5 M H₂SO₄), and the plate was gently agitated for 5 seconds. Optical density was assessed at 450 nm utilizing a microplate reader (TECAN, Männedorf, Switzerland), and insulin concentrations were determined.

Serum fructosamine was quantified using a colorimetric assay based on the reduction of nitroblue tetrazolium on a Biosystems BA400 clinical chemistry analyzer (Biosystems S.A., Barcelona, Spain). Plasma glucose was quantified using the glucose oxidase-peroxidase (GOD-POD) method on a Biosystems BA400 clinical chemistry analyzer, with quinoneimine assessed at 510 nm. Serum lipids were measured via an Automated Clinical Chemistry Analyzer (Biosystems BA400). TC was quantified using the cholesterol oxidase-peroxidase (CHOD-PAP) technique. TGs were measured by a colorimetric enzymatic test using glycerol-3-phosphate oxidase-peroxidase (GPO-POD) method, LDL-C and HDL-C were measured using a homogeneous assay method.

### DNA extraction and 16S rRNA gene sequencing

Fecal DNA was extracted with the ZymoBIOMICS™ DNA Miniprep Kit (Zymo Research Corporation, CA, USA) in accordance with the manufacturer’s instructions. Briefly, 200 mg of feces was added to a ZR BashingBead™ Lysis Tube with 750 μL of lysis solution, then homogenized using a bead beater. Lysates were centrifuged at ≥10,000 × g for 1 minute, and up to 400 μL of supernatant was transferred to a Zymo-Spin™ III-F filter, followed by centrifugation at 8,000 × g for 1 minute. The filtrate was combined with 1,200 μL of DNA binding buffer, and 800 μL of this mixture was applied twice to a Zymo-Spin™ IICR column, with each application followed by centrifugation at 10,000 × g for 1 minute. The column was progressively washed with 400 μL of DNA wash buffer 1, followed by 700 μL of DNA wash buffer 2, and concluded with a final wash of 200 μL of DNA wash buffer 2. DNA was eluted by adding 80 μL of DNase/RNase-free water to the column, incubating for 1 minute, and centrifuging at 10,000 × g. To eliminate inhibitors, 600 μL of HRC preparation solution was processed through a Zymo-Spin™ III-HRC filter, and the eluted DNA was further filtered through the same column by centrifugation at 16,000 × g for 3 minutes. The purified DNA was subsequently prepared for downstream 16S rRNA gene sequencing. The integrity of DNA samples was assessed by 2% agarose gel electrophoresis. The V4 region of the 16S rRNA gene was amplified using 515F/806R 16S rRNA primers (Novogene Co., Ltd., Beijing, China). The libraries were sequenced on an Illumina paired-end platform (Illumina, San Diego, CA, USA) to produce 300 bp paired-end raw reads, following the manufacturer’s guidelines. All PCR amplification and sequencing procedures were conducted at Novogene Co., Ltd. (Beijing, China) utilising an Illumina NovaSeq 6000 platform. Sequence reads were processed using the Quantitative Insights Into Microbial Ecology 2 (QIIME2) pipeline (version 2022.8). Quality filtering, denoising, and chimera removal were performed using the DADA2 plugin, which includes error correction and removal of low-quality sequence variants [36,37]. Sequences were truncated and filtered based on quality scores prior to denoising. Amplicon sequence variants (ASVs) were inferred and used for downstream analysis. Low-abundance features were filtered where appropriate to reduce noise in the dataset. Taxonomic assignment of the 16S rRNA sequences was conducted using the Silva 138 99% taxonomy classifier [38,39]. ASVs were aligned using the MAFFT plugin in QIIME2.

### Statistical analyses

Descriptive statistics for fGCM, MDA, fructosamine, insulin, glucose, triglycerides, cholesterol, LDL, HDL, and relative abundance of fecal microbiota are reported as mean ± standard error of the mean (SEM). Statistical analyses were conducted with R software (version 4.4.1) [40]. Differences in biomarker concentrations among dogs in the three shelters were initially analyzed using one-way analysis of variance (ANOVA). Residuals from the ANOVA were tested for normality using the Shapiro–Wilk test [41], and for homogeneity of variances using Levene’s test. If both assumptions were met, ANOVA results were considered valid and followed by Tukey’s Honestly Significant Difference (HSD) test for post hoc comparisons (e.g., fructosamine, glucose, HDL). If the assumptions were violated (e.g., fGCM, MDA, insulin, triglyceride, cholesterol, LDL), non-parametric Kruskal–Wallis tests were used instead. Subsequent pairwise comparisons were conducted using the Wilcoxon rank-sum test, with p-values adjusted using the Benjamini–Hochberg method. Statistical significance was defined as *p* < 0.05. Alpha diversity indicators, including Observed characteristics, Shannon entropy, and Pielou evenness, were computed to evaluate microbiological variety. Comparisons of alpha diversity among groups were conducted utilizing the Kruskal–Wallis test. Microbiome data analysis was conducted using QIIME2. Relative abundance data at the phylum, family, and genus levels were compared across shelters using the Kruskal–Wallis test followed by Dunn’s post hoc correction. The relative abundances of microbial taxa were expressed as percentages. The Bray-Curtis, Jaccard, Unweighted UniFrac, and Weighted UniFrac distance matrices were employed for beta diversity analysis, with findings visualised via principal coordinate analysis (PCoA) in R software version 4.4.1. Permutational Multivariate Analysis of Variance (PERMANOVA) was employed to statistically assess significant variations in microbial composition between the three shelters.

## 3. Results

### Physiological and metabolic parameters

Significant differences in physiological and metabolic markers were observed across the three shelters (Table 2). The fGCM concentrations were highest in Shelter C, followed by Shelter B and then Shelter A; all pairwise comparisons were statistically significant (*p* < 0.05). For MDA, no significant differences were detected among the shelters, with values ranging from 7.62 ± 0.59 to 9.39 ± 0.51 µM (*p* = 0.0936). In contrast, fructosamine concentrations varied significantly, being lowest in Shelter B and highest in Shelter C. Insulin concentrations showed marked differences, with Shelter B presenting the highest concentration, followed by Shelter C and then Shelter A. Glucose concentrations were elevated in Shelter B compared to Shelters A and C, which did not differ significantly from each other. Triglyceride concentrations were higher in Shelter C than Shelter B, while Shelter A did not differ significantly from the others. In contrast, cholesterol concentrations did not differ among the shelters, although the result approached statistical significance (*p* = 0.0502). LDL concentrations were higher in Shelter B, followed by Shelter C and Shelter A. In contrast, HDL was highest in Shelter C and lowest in Shelter B, with all pairwise comparisons being statistically significant.

**Table 2:** Mean (± SEM) and range of fGCM, MDA, and metabolic parameters in fecal samples from dogs housed in three shelters in Thailand.

Parameters	Shelter A (n = 300)	Shelter B (n = 300)	Shelter C (n = 300)	*p* value
fGCM (ng/g)	207.41 ± 4.55^a^ (92.57–697.99)	224.55 ± 4.16^b^ (81.70–561.97)	278.71 ± 10.44^c^ (20.88–1420.00)	<0.001
**Parameters**	**Shelter A** **(n = 60)**	**Shelter B** **(n = 60)**	**Shelter C** **(n = 60)**	***p* value**
MDA (µM)	7.80 ± 0.41 (1.89–15.66)	9.39 ± 0.51 (1.51–16.79)	7.62 ± 0.59 (0.94–16.79)	0.0936
Fructosamine (mM)	1.29 ± 0.02^b^ (0.99–1.68)	1.11 ± 0.02^a^ (0.74–1.56)	1.38 ± 0.02^c^ (0.96–1.83)	<0.001
Insulin (μg/L)	736.63 ± 92.32^a^ (ND – 4,255.00)	1,665.30 ± 188.28^c^ (121.00–7,169.00)	1,010.40 ± 72.47^b^ (ND – 2,280.00)	<0.001
Glucose (mg/dL)	63.00 ± 2.50^a^ (21.00–135.00)	78.27 ± 2.68^b^ (28.00–126.00)	58.72 ± 1.90^a^ (21.00–94.00)	<0.001
Triglyceride (mg/dL)	95.45 ± 6.33^ab^ (11.00–256.00)	77.48 ± 4.21^a^ (28.00–214.00)	101.33 ± 6.62^b^ (17.00–315.00)	0.0073
Cholesterol (mg/dL)	179.53 ± 5.80 (92.00–363.00)	188.13 ± 5.79 (115.00–354.00)	195.88 ± 4.77 (114.00–283.00)	0.0502
LDL (mg/dL)	4.75 ± 0.52^a^ (ND – 23.00)	14.28 ± 1.90^c^ (1.00–67.00)	6.18 ± 0.38^b^ (ND – 15.00)	<0.001
HDL (mg/dL)	141.40 ± 3.78^b^ (74.00–198.00)	129.00 ± 3.37^a^ (83.00–193.00)	158.93 ± 3.41^c^ (92.00–217.00)	<0.001

fGCM = fecal glucocorticoid metabolites; MDA = malondialdehyde; LDL = low-density lipoprotein; HDL = high-density lipoprotein; ND = nondetectible

a,b,c Means with different letters are significantly different (*p* < 0.05)

### Fecal microbial diversity

**Fig 1:**
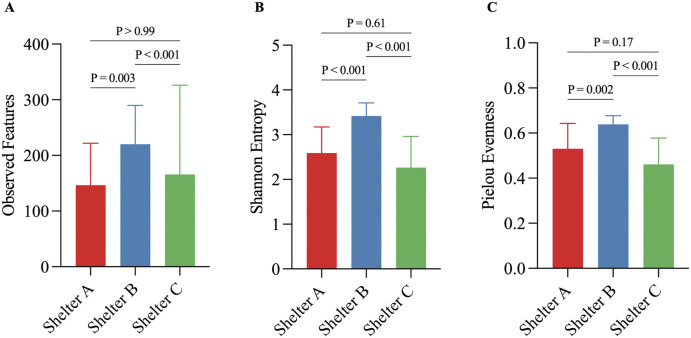
Alpha diversity of fecal microbiota in fecal samples from dogs housed in three shelters in Thailand. Diversity metrics include **(A)** Observed Features (richness), **(B)** Shannon Entropy (richness and evenness), and **(C)** Pielou Evenness (distribution uniformity). Statistical significance was determined using the Kruskal–Wallis test followed by Dunn’s post hoc comparison (*p* < 0.05).

**Fig 2:**
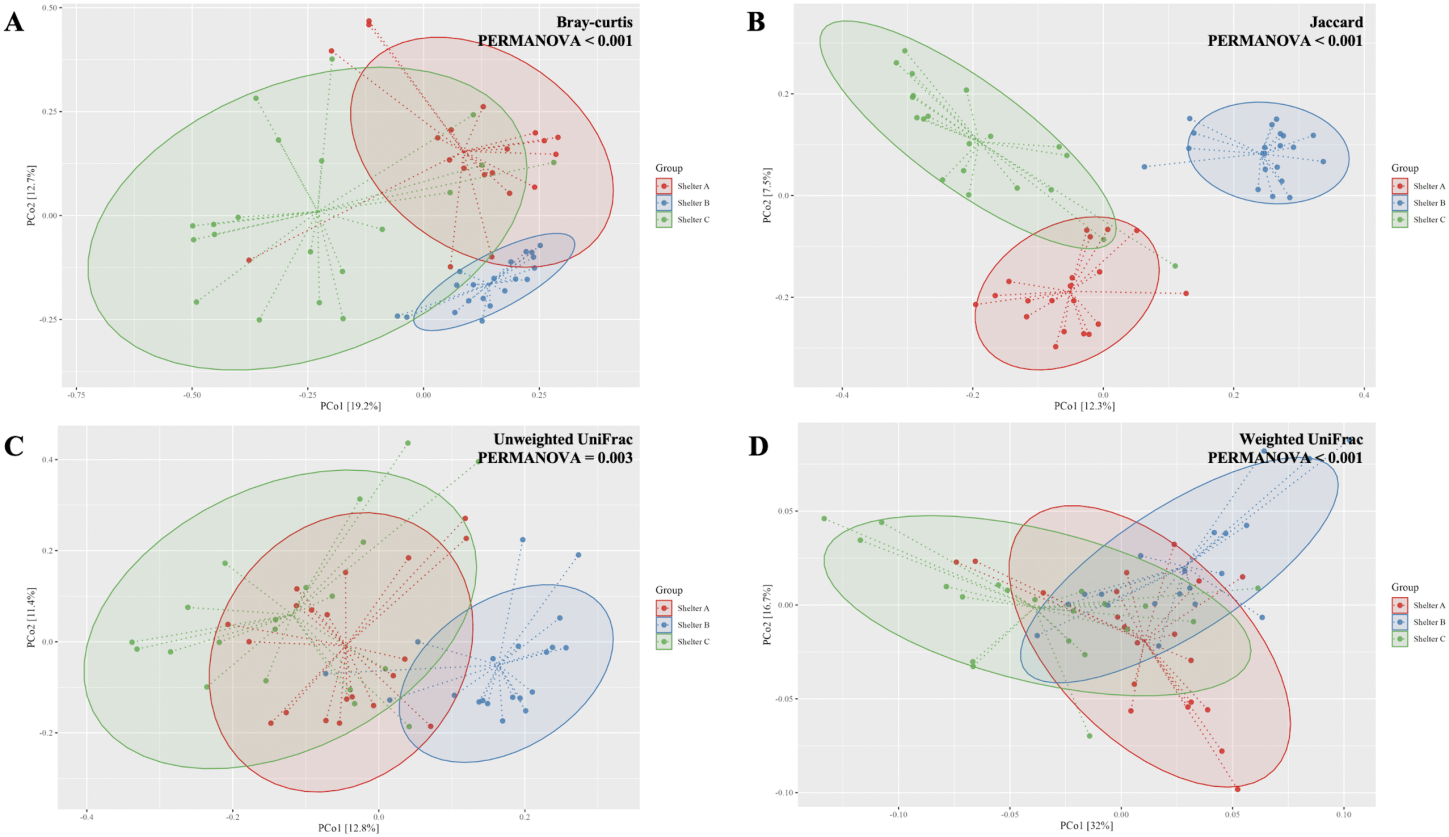
Principal Coordinates Analysis of beta diversity showing microbiota composition in fecal samples from dogs housed in three shelters in Thailand. Each point represents an individual sample, and clustering reflects similarities in microbial community structure. Distinct clustering by shelter indicates that dogs from each facility harbored compositionally different fecal microbiota. ‌‌Statistical significance was assessed using PERMANOVA (*p* < 0.05).

Alpha diversity metrics revealed significant differences in microbial richness and evenness among dogs from the three shelters (Fig 1). Dogs in Shelter B had the highest number of observed features (ASVs), greater than Shelter A (*p* = 0.003) and Shelter C (*p* < 0.001), while no differences were observed between Shelters A and C (*p* > 0.99). Shannon entropy, reflecting overall diversity, was also highest in Shelter B (*p* < 0.001), with no difference between Shelters A and C (*p* = 0.61). Pielou evenness followed a similar pattern with Shelter B having higher evenness than Shelter A (*p* = 0.002) or Shelter C (*p* < 0.001), with no difference between Shelters A and C (*p* = 0.17). Beta-diversity analysis using Principal Coordinates Analysis (PCoA) revealed distinct clustering of microbial communities across shelters (Fig 2). Bray–Curtis dissimilarity revealed compositional separation, with Shelter B clustering distinctly from Shelters A and C (PERMANOVA, *p* < 0.001). Similar patterns were observed with Jaccard distance (*p* < 0.001), Unweighted UniFrac (*p* = 0.003), and Weighted UniFrac (*p* < 0.001), confirming both taxonomic and phylogenetic differences. These findings suggest that shelter-specific conditions strongly influence fecal microbiota composition (Fig 2).

**Fig 3:**
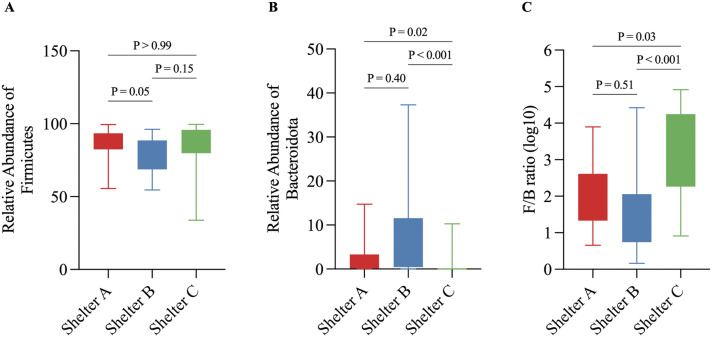
Relative abundance of major bacterial phyla and Firmicutes-to-Bacteroidota (F/B) ratio in fecal samples from dogs housed in three shelters in Thailand. **(A)** Firmicutes abundance did not differ significantly across shelters. **(B)** Bacteroidota abundance was greatest in Shelter B and lowest in Shelter C (*p* < 0.05). **(C)** The F/B ratio (log₁₀-transformed) was significantly higher in Shelter C, suggesting differences in microbial composition. Statistical comparisons were conducted using the Kruskal–Wallis test with Dunn’s post hoc correction.

**Fig 4:**
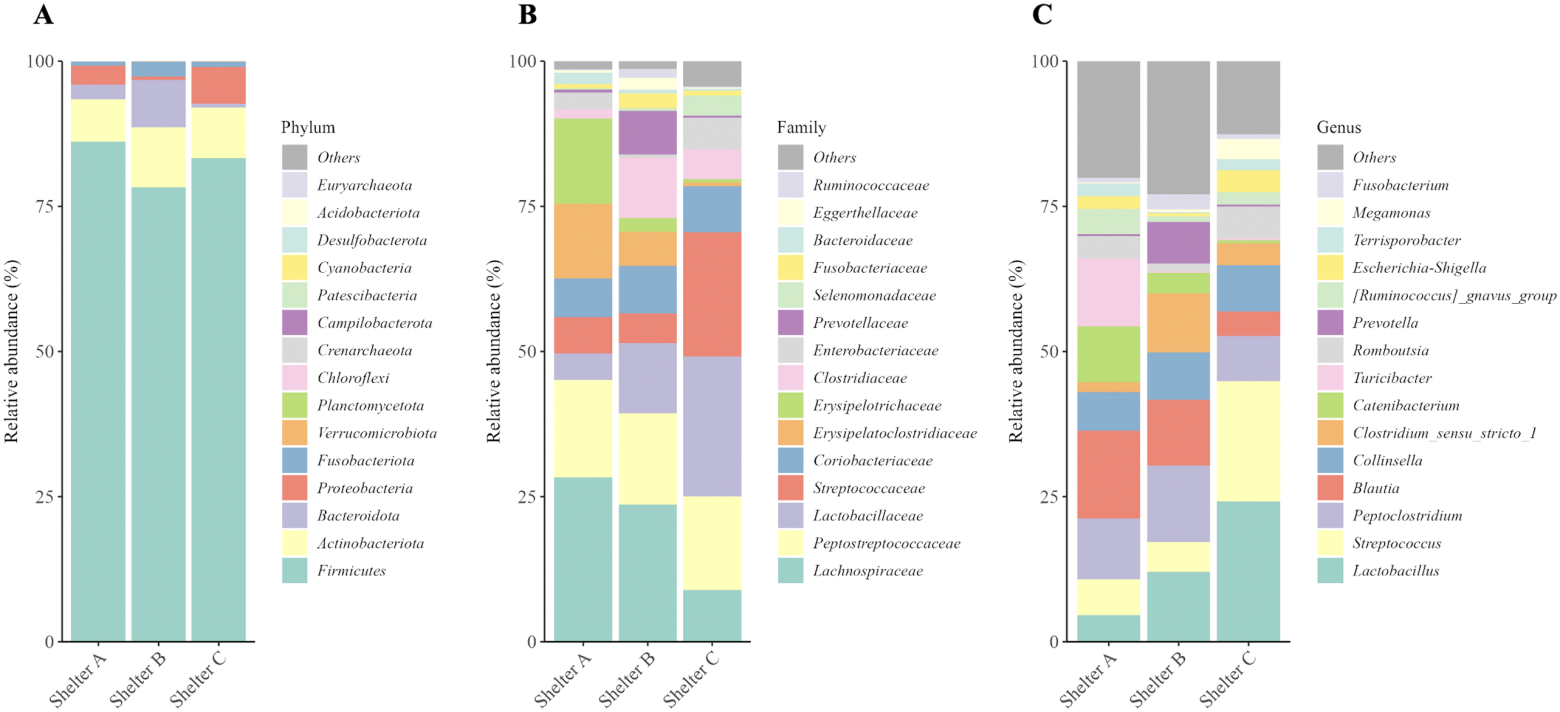
Taxonomic composition of fecal microbiota in dogs housed in three shelters in Thailand at the (A) phylum, (B) family, and (C) genus levels, based on 16S rRNA gene sequencing. At the phylum level, Firmicutes was the most dominant across all shelters, followed by Actinobacteriota, Bacteroidota, Proteobacteria, and Fusobacteriota. Relative abundance patterns varied among shelters, reflecting differences in environmental and management conditions.

At the phylum level, Firmicutes dominated across all shelters, comprising over 78% of the total bacterial population. Shelter A showed the highest Firmicutes abundance, followed by Shelter C and Shelter B. In contrast, Bacteroidota was most abundant in Shelter B, while it was markedly lower in Shelters A and C. At the family level, Lachnospiraceae was most prevalent in Shelter A and least in Shelter C. Lactobacillaceae and Streptococcaceae were notably more abundant in Shelter C than in the other shelters. At the genus level, *Lactobacillus* and *Streptococcus* followed the same trend, being more abundant in Shelter C than in Shelters A or B. The genus *Blautia* was most common in Shelter A and least in Shelter C (Table 3, Fig 3). The relative abundances of Firmicutes and Bacteroidota, along with the F/B ratio, varied significantly across shelters. Firmicutes dominated across all shelters, with Shelter A showing higher levels than Shelter B. Bacteroidota was more abundant in Shelter B than in Shelters A and C (*p* < 0.001). Consequently, the F/B ratio was highest in Shelter C, followed by Shelter A and then Shelter B (Table 4, Fig 4).

**Table 3:** Mean relative abundance (%) of the five most common bacterial taxa at the phylum, family, and genus levels in fecal samples from dogs housed in three shelters in Thailand.

Taxa	Shelter A (n = 20)	Shelter B (n = 20)	Shelter C (n = 20)	*p* value
**Phyla**				
Firmucutes	86.14 ± 2.78 (55.55–99.48)	78.21 ± 2.53 (54.45–96.23)	83.29 ± 3.87 (33.93–99.50)	0.0594
Actinobacteriota	7.27 ± 1.41 (0.14–25.19)	10.32 ± 1.20 (2.95–26.73)	8.69 ± 2.42 (0.01–45.67)	0.1123
Bacteroidota	2.53 ± 0.85^a^ (0.00–14.60)	8.20 ± 2.32^b^ (0.00–37.08)	0.69 ± 0.51^a^ (0.00–10.15)	<0.001
Proteobacteria	3.27 ± 2.09^a^ (0.01–35.62)	0.56 ± 0.21^a^ (0.01–4.38)	6.29 ± 3.24^b^ (0.09–64.74)	0.0006
Fusobacteriota	0.73 ± 0.38^a^ (0.00–6.76)	2.61 ± 0.80^b^ (0.00–13.17)	0.87 ± 0.68^a^ (0.00–13.81)	0.0158
Other	0.05 ± 0.02 (0.00–0.50)	0.10 ± 0.08 (0.00–1.58)	0.16 ± 0.11 (0.00–2.19)	0.2436
**Family**				
Lachnospiraceae	28.33 ± 4.28^b^ (0.56–64.59)	23.66 ± 1.79^b^ (7.81–38.30)	8.91 ± 3.31^a^ (0.01–49.17)	0.0001
Peptostreptococcaceae	16.73 ± 3.27 (2.83–65.76)	15.69 ± 1.29 (7.79–28.31)	16.12 ± 5.09 (0.02–95.88)	0.1503
Lactobacillaceae	4.55 ± 2.18^a^ (0.00–41.06)	12.03 ± 2.60^b^ (0.26–42.95)	24.15 ± 6.35^b^ (0.02–99.12)	0.0019
Streptococcaceae	6.24 ± 2.80^a^ (0.05–42.14)	5.12 – 0.94^a^ (0.10–13.33)	21.31 ± 5.07^b^ (0.02–84.03)	0.0225
Coriobacteriaceae	6.63 ± 1.27 (0.09–20.93)	8.16 ± 0.92 (2.22–19.98)	7.93 ± 2.28 (0.00–43.44)	0.3284
Other	37.52 ± 5.71 (2.12–89.94)	35.32 ± 2.66 (20.36–58.56)	21.57 ± 3.87 (0.74–68.87)	0.2769
**Genera**				
*Lactobacillus*	4.55 ± 2.22^a^ (0.00–41.05)	12.04 ± 2.60^b^ (0.26–42.94)	24.15 ± 6.35^b^ (0.01–99.11)	0.0018
*Streptococcus*	6.23 ± 2.80^a^ (0.05–42.13)	5.10 ± 0.94^a^ (0.09–13.30)	20.69 ± 4.97^b^ (0.02–83.52)	0.0260
*Peptoclostridium*	10.47 ± 1.81^b^ (0.43–27.58)	13.14 ± 0.99^b^ (6.00–23.47)	7.83 ± 2.00^a^ (0.01–24.25)	0.0411
*Blautia*	15.06 ± 2.71^b^ (0.10–35.99)	11.31 ± 0.76^b^ (2.89–16.82)	4.23 ± 1.65^a^ (0.00–30.05)	0.0002
*Collinsella*	6.63 ± 1.27 (0.09–20.93)	8.16 ± 0.92 (2.22–19.98)	7.93 ± 2.28 (0.00–43.45)	0.3284
Other	57.06 ± 5.27 (16.42–97.24)	50.25 ± 2.77 (31.05–74.67)	35.15 ± 5.41 (0.76–99.88)	0.0533

a,b,c Means with different letters are significantly different (*p* < 0.05)

**Table 4:** Relative abundance of the Firmicutes and Bacteroidota phyla, and the Firmicutes-to-Bacteroidota (F/B) ratio, in fecal samples from dogs housed in three shelters in Thailand.

Group	Firmicutes (%)	Bacteroidota (%)	F/B ratio
**Shelter A** (n = 20)	86.14	2.53	34.05
**Shelter B** (n = 20)	78.21	8.20	9.53
**Shelter C** (n = 20)	83.29	0.69	120.71

## 4. Discussion

This study examined the influence of long-term shelter conditions, including space allocation, enrichment, and management practices, on indicators of stress, metabolism, and fecal microbiota in shelter dogs. Although not all parameters differed significantly across shelters, several trends suggest that management conditions play an important role in shaping canine welfare. Dogs housed in Shelter C, characterized by limited space and absence of enrichment, showed higher mean fGCM and fructosamine concentrations and an imbalanced F/B ratio, suggesting potential shifts in microbial composition associated with stress and metabolic disturbance. In contrast, dogs from Shelter A, which provided more space and structured enrichment, exhibited lower fGCM concentrations and more normal metabolic and microbial values. Interestingly, dogs in Shelter B exhibited higher microbial diversity despite moderate crowding, a pattern that differs from that observed in the other shelters. This outcome may reflect unmeasured factors such as differences in diet composition, enclosure design, or social structure that could influence microbial stability and metabolic responses. Further research is needed to clarify these relationships. It should be noted that fecal microbiota analysis in this study was based on 16S rRNA gene sequencing, which primarily provides taxonomic information and does not directly capture functional or metabolic activity of the microbial community. Therefore, interpretations related to microbial function should be made with caution. Overall, these findings reinforce the complexity of welfare determinants in shelter environments.

### fGCM concentrations

This study showed the influence of housing conditions, particularly space per dog, enrichment, and overall management quality, on fGCM and physiological stress in shelter dogs. Among the three shelters, dogs in Shelter A, which offered the largest living space (20.3 m² per dog), regular enrichment, and consistent human interaction, exhibited the lowest fGCM concentrations. These findings align with previous studies indicating that enriched environments and adequate space are associated with HPA axis regulation and lower cortisol concentrations in dogs [42,43]. In contrast, Shelter C, characterized by the most crowded conditions (3.0 m² per dog), absence of enrichment, and limited human interaction, showed significantly higher fGCM concentrations. The association between space, enrichment, and stress was further supported by our previous study involving eight dog shelters in Thailand, three of which were included in the present analysis. That study found that shelters providing more space and environmental stimulation (such as Shelter A) were associated with lower barking frequencies [31], a common behavioral marker of canine stress [13], and better body condition scores. By contrast, Shelters B and C scored higher in the Reaction Toward Human (RTH) assessment, reflecting greater unfamiliarity and increased aggression toward strangers, both of which are commonly associated with prolonged stress. Thus, suboptimal housing can lead to both physical and psychological stress, underscoring the importance of multidimensional welfare assessments.

Dogs in this study had been housed at the shelters for more than 1 year (ranging from 1–5 years), a duration typically associated with chronic stress [44,45]. Differences in physiological stress levels among shelters suggest that environmental conditions play a critical buffering role. Previous studies have shown that prolonged confinement in shelters, particularly under crowded or barren conditions, can lead to chronic activation of the HPA, resulting in elevated glucocorticoid concentrations and associated health risks [46,47]. This aligns with findings from Raudies et al. [44] that reported dogs housed in Austrian no-kill shelters for over a year exhibited more aggression, higher arousal, and increased stress-related behaviors compared to short-term residents. However, the present study found that dogs in Shelter A, despite also being long-term residents, exhibited the lowest fGCM concentrations. This suggests that factors such as larger space per dog, access to enrichment, and regular human interaction can mitigate the physiological impacts of long-term shelter housing. By contrast, Shelter C, which offered the least space and lacked enrichment, showed the highest fGCM concentrations, consistent with chronic stress exposure. These findings extend prior research by demonstrating that chronic stress is not solely a function of housing duration but may also be influenced by the quality of the shelter environment. Appropriate environmental management, including space, stimulation, and social interaction, can serve as protective factors that reduce the burden of long-term confinement on canine welfare [8,10].

### Metabolic, oxidative stress, and lipid biomarkers

In addition to stress hormone profiles, several metabolic parameters varied significantly across shelters, offering further insight into how environmental conditions influence physiological regulation in dogs. Fructosamine, insulin, and glucose are interrelated markers that reflect different aspects of glycemic metabolism. Measures of glucose indicate short-term fluctuations in blood sugar, while insulin reflects the body’s regulatory effort to maintain glycemic homeostasis [48–50]. Fructosamine reflects average blood glucose over the preceding 1–3 weeks and thus serves as an indicator of more chronic glycemic status [51]. Dogs in Shelter A exhibited the lowest insulin concentrations, while glucose concentrations were lower in Shelters A and C than in Shelter B, with no significant difference between Shelters A and C, suggesting relatively more stable metabolic regulation in Shelter A. This may be partly influenced by dietary factors, as Shelter A not only provided commercial dry and canned food but also occasionally supplemented meals with cooked meat, such as boiled chicken breast. By contrast, to reduce costs, Shelter C frequently provided rice-based meals, which may have resulted in less balanced glycemic responses. Fructosamine concentrations were highest in Shelter C and lowest in Shelter B, with Shelter A levels in the middle; however, all were within the normal range for nondiabetic domestic dogs [52].

Fructosamine is formed via the non-enzymatic binding of glucose to serum proteins, particularly albumin, so its concentration may be influenced by nutritional status and protein levels [53]. Dogs in Shelter A had better body condition [31] and intermediate fructosamine concentrations with normoglycemia. This may be attributed to improved nutritional status, as higher serum protein and albumin concentrations, commonly seen in healthier animals, can influence fructosamine independently of glucose levels [54,55]. Additionally, mild postprandial glycemic fluctuations, undetectable by single-point glucose measurements, may contribute to higher fructosamine values, such as dogs in Shelter A, who received more consistent diets that could lead to subtle elevations in postprandial glucose without causing overt hyperglycemia [56]. This pattern is consistent with findings in diabetic dogs, laboratory animals, and livestock [20,57,58], although there was no evidence that any of the dogs in this study were diabetic. Shelter C showed the highest fructosamine concentrations, which may indicate moderate alterations in glucose metabolism. These conditions are known to elevate stress hormones such as cortisol, epinephrine, and glucagon, which stimulate hepatic glucose production and impair peripheral glucose uptake, leading to sustained hyperglycemia even in non-diabetic animals [59,60]. Meanwhile, Shelter B had relatively higher glucose and insulin concentrations, which may reflect stress-related alterations in glycemic regulation or early metabolic imbalance [61]. High-carbohydrate rice diets in some shelters could have promoted postprandial glucose spikes and increased insulin demand, potentially leading to impaired glycemic control over time [56]. These findings agree with previous reports showing that chronic stress impairs glucose metabolism and disrupts endocrine function in dogs [42,62,63].

There were no differences among shelters in MDA concentrations; however, several lipid parameters did vary. While total cholesterol did not differ significantly, triglycerides, LDL, and HDL concentrations showed notable variation. These lipids are key components in energy storage, hormone production, and inflammation regulation [64]. Slightly higher triglyceride levels observed in Shelter C may reflect minor variations related to dietary composition or activity levels, rather than stress-induced lipolysis [65]. Chronic activation of the HPA, common under prolonged stress, increases glucocorticoids, which can stimulate hepatic triglyceride synthesis and signal early metabolic strain [66,67]. Shelters B and C exhibited higher LDL concentrations and a trend toward elevated total cholesterol compared to Shelter A. This may suggest impaired lipid clearance or increased hepatic cholesterol production, potentially driven by chronic stress or subclinical inflammation [68]. Persistent elevation of LDL is associated with systemic inflammation and oxidative stress, contributing to long-term metabolic dysfunction and cardiovascular risk [69,70], as reported in dogs [71]. Likewise, animal models of chronic stress demonstrate increased LDL atherogenicity through inflammation and oxidative damage [72]. HDL concentrations also differed among shelters and can be influenced by multiple factors, including diet, inflammation, and stress responses [73,74]. In dogs, HDL levels may reflect underlying metabolic status and inflammatory conditions, which can be altered by physiological stress [75]. While higher HDL is typically considered protective, its function may be compromised under chronic stress or inflammatory conditions. In such contexts, HDL particles may lose their antioxidant and anti-inflammatory properties or reflect a compensatory response to increased oxidative stress [75,76]. Therefore, elevated HDL concentrations in some shelters should be interpreted cautiously, as they may indicate physiological stress rather than better welfare.

### Fecal microbial analysis

To provide a descriptive overview of microbial patterns among the shelters, we examined both microbial diversity and taxonomic composition. Shelter A appeared to provide the most favorable welfare conditions, including low dog density, consistent management, and better overall nutritional support that resulted in better body condition. It also exhibited the lowest physiological stress markers, with significantly lower fGCM concentrations. However, despite these advantages, dogs in Shelter A had the lowest gut microbial diversity across all alpha diversity indices. The microbiota was dominated by Firmicutes and Actinobacteriota, particularly Lactobacillaceae and Streptococcaceae (Fig 4), taxa commonly linked to better gut health [77]. Nonetheless, the low diversity may suggest reduced microbial resilience or limited environmental microbial exposure [78]. In contrast, Shelter B, which had moderate dog density, mixed feeding practices, and elevated fGCM, glucose, and insulin, showed the highest alpha diversity. The fecal microbiota featured a wider range of taxa, including *Prevotella*, *Blautia*, and *Collinsella*, involved in carbohydrate fermentation and SCFA production [79,80]. The combination of microbial richness and elevated stress markers suggests this diversity may reflect adaptive microbial shifts rather than optimal host health [78]. Dogs in Shelter C, with the highest dog density and limited nutritional quality with minimal protein, showed intermediate richness, and the lowest Shannon entropy and evenness, reflecting reduced microbial stability. Elevated levels of Proteobacteria and *Escherichia-Shigella* in those dogs may reflect microbial imbalance associated with stress and suboptimal environmental conditions [81]. These findings agree with previous research in dogs and other species, which has shown that reduced alpha diversity is frequently linked to chronic stress and diminished welfare. For example, dogs carrying extended-spectrum β-lactamase (ESBL)-producing bacteria, which are commonly found in animals living in crowded and stressful shelter environments, have significantly lower gut microbial diversity compared to non-carriers [82]. Similarly, mice subjected to chronic stress showed reduced alpha diversity and could transmit depressive-like behavior via fecal transplants [83]. In humans, high stress levels were also associated with reduced diversity and altered gut microbiota [84]. Together, these results indicate that gut microbial diversity and composition do not always align linearly with conventional welfare indicators. While Shelter A provided the best physical and physiological welfare, its microbiota showed the lowest diversity. Shelter B, with moderate stress and dietary variation, had the richest microbial community, whereas Shelter C’s unfavorable conditions were reflected in signs of microbial instability. These findings demonstrate the complex, multifactorial nature of fecal microbiota and the need to interpret it in conjunction with both environmental and physiological parameters.

Beta diversity in gut microbiota is influenced by factors such as breed, age, diet, health, and stressors like overcrowding and inconsistent care [82,85,86]. In this study, distinct clustering by shelter indicated significant differences in microbial profiles, likely reflecting welfare and environmental variation. Shelter A showed moderate clustering and lower alpha diversity. This pattern may reflect a stable but less environmentally complex microbial community. Importantly, Shelter A also exhibited the lowest fGCM concentrations, supporting the idea that low physiological stress promotes microbial uniformity and resilience, albeit with reduced diversity [29,87,88]. Shelter B showed tight clustering with the highest alpha diversity, likely due to moderate enrichment, mixed diets, and social interactions. This suggests that balanced variability can foster microbial richness while maintaining community stability [29]. In such contexts, diversity may reflect healthy niche expansion rather than dysbiosis [78], aligning with ecological theory that moderate environmental complexity supports diversity without destabilizing microbial communities [89]. In contrast, Shelter C exhibited dispersed clustering, low evenness, and elevated levels of Proteobacteria and *Escherichia-Shigella* patterns linked to microbial instability and dysbiosis. Combined with the highest fGCM and suboptimal diets, these findings suggest that chronic stress and environmental unpredictability likely disrupted gut microbiota [90]. Overall, these results demonstrate the influence of shelter conditions on gut microbiota, with both stress and environmental quality shaping microbial diversity and stability [91–93].

Although Firmicutes was the predominant phylum across all shelters, the F/B ratio varied substantially across them. A higher F/B ratio has been associated with shifts in microbial composition and has been linked to chronic stress, metabolic dysfunction, increased inflammation, and impaired gut health [94,95]. The microbial profiles in this study suggest that the gut microbial imbalance observed in Shelter C may reflect compromised physiological and welfare status.

Analysis of gut microbial composition at the phylum level in this study revealed that the five most abundant phyla were Firmicutes, Actinobacteriota, Bacteroidota, Proteobacteria, and Fusobacteriota. This profile is consistent with previous studies in dogs [96,97]. However, notable differences emerge when compared to other species. For instance, in humans, Verrucomicrobia is often more prominent than Fusobacteriota [98], while in elephants, Spirochaetes or Verrucomicrobia tend to dominate over Fusobacteriota [99]. Nevertheless, the relative abundance and presence of the other four major phyla remain largely similar across species. At the genus level, dogs in Shelter A exhibited a higher abundance of beneficial taxa such as *Blautia*, a known producer of short-chain fatty acids (SCFAs) with anti-inflammatory properties [100]. The presence of SCFA-producing genera in both Shelters A and B indicates a potentially healthier colonic environment and greater anti-inflammatory capacity. In contrast, dogs in Shelter C had elevated levels of *Lactobacillus* and *Streptococcus*. While these genera are typically considered beneficial or probiotic [101], their disproportionate abundance may represent an adaptive response to stress or underlying microbial imbalance. Such overrepresentation has been associated with compromised gut barrier integrity and a heightened risk of inflammation. For example, a study by Liang et al. [102], reported that *Lactobacillus* overgrowth may serve as a compensatory mechanism during microbial imbalance, as observed in patients with immune-mediated necrotizing myopathy. The same study also demonstrated that in dogs, stress-induced dysbiosis could drive *Lactobacillus* proliferation as an adaptive response aimed at modulating gut-brain axis signaling.

### Limitations

While this study provides valuable insights into the physiological and microbiological responses of dogs housed under different shelter conditions, several important limitations must be acknowledged. The lack of standardized diets among shelters represents a major confounding factor and is likely one of the most important sources of variation influencing both metabolic and microbiome outcomes. Differences in dietary composition, feeding frequency, and nutrient balance across shelters may have substantially contributed to the observed physiological and microbial differences. Therefore, the effects of shelter conditions should be interpreted with caution, as dietary factors cannot be fully separated from environmental influences in this study. In addition, age and breed composition were not standardized across shelters, which may have contributed to variability in physiological and microbiome outcomes. These demographic factors are known to influence metabolic parameters, stress responses, and gut microbial composition, and therefore represent additional sources of variation that could not be fully controlled in this study. The cross-sectional design of this study prevents causal interpretation; therefore, the observed relationships between shelter conditions and physiological and microbiological outcomes should be interpreted as associations rather than causal effects. While sample sizes were sufficient for primary comparisons, subtle effects may have gone undetected. In addition, the relatively small subsample size for fecal microbiome analysis (n = 20 per shelter) may limit statistical power and reduce representativeness compared to the larger physiological dataset. Therefore, microbiome findings should be interpreted with appropriate caution. It should be noted that statistical analyses of relative abundance were performed using non-parametric methods that do not fully account for the compositional nature of microbiome data. Therefore, results should be interpreted with caution, and future studies using compositional-aware methods may provide more robust insights. Moreover, fecal microbiota composition primarily reflects short-term or localized physiological states and may not reflect the entire composition of the gastrointestinal tract.

## 5. Conclusions

This study supports and expands earlier research by Saengthong et al. [31], who found that lower disease prevalence and reduced stress-related behaviors in shelter dogs were associated with more structured management and increased space per dog. By incorporating physiological indicators such as analyses of fGCM, oxidative stress markers, metabolic profiles, and fecal microbiota, this study further confirms that environmental and management factors substantially influence the welfare of long-term shelter dogs. Animals in well-managed shelters with ample space exhibited lower stress hormone levels, more stable metabolic profiles, and greater microbial diversity and balance. In contrast, dogs in overcrowded and informally managed shelters, such as Shelter C, a temple-based model common in Thailand, showed signs consistent with chronic stress and microbial dysbiosis. These findings underscore the importance of evidence-based shelter practices. Elements such as adequate space, predictable routines, environmental enrichment, and trained staff not only affect observable behavior but also exert measurable physiological effects. Incorporating animal welfare science into shelter management can enhance well-being, reduce healthcare costs, and potentially improve adoption outcomes. Non-invasive physiological indicators provide valuable tools for routine welfare monitoring and informed decision-making. Future studies should employ longitudinal or intervention-based designs to clarify causal relationships and track changes over time. Including additional biomarkers, such as immunoglobulin A [103], and functional microbiome analyses (e.g., metagenomics, SCFA profiling) [104], would provide a more comprehensive and mechanistic understanding of how shelter environments shape welfare outcomes. Collectively, this and previous research provide compelling evidence for the systematic improvement of shelter conditions to promote sustainable, high-welfare outcomes for dogs.

## Supporting information

S1 File Raw fecal glucocorticoid metabolite (fGCM) concentrations in dogs across three shelters. This file contains the raw fecal glucocorticoid metabolite (fGCM) concentration data collected from dogs housed in three shelters (Shelter A, Shelter B, and Shelter C). A total of 900 fecal samples are included (300 samples per shelter).
(XLSX)

S2 File Raw biomarker data of dogs across three shelters. This file contains the raw biomarker data collected from dogs housed in three shelters (Shelter A, Shelter B, and Shelter C). A total of 180 samples are included (60 samples per shelter). Biomarkers include malondialdehyde (MDA), glucose, fructosamine, cholesterol, triglycerides, high-density lipoprotein (HDL), low-density lipoprotein (LDL), and insulin.
(XLSX)

S1 Table Sample metadata and NCBI Sequence Read Archive (SRA) accession numbers for all fecal samples included in this study. (DOCX)
