# Supplementary material for: Linking shelter conditions to health: A multisystem analysis of stress, metabolism, and fecal microbiota in dogs
Source: PLoS One. 2026 Jul 1;21(7):e0350401. doi: 10.1371/journal.pone.0350401 (PMC13322536; doi:10.1371/journal.pone.0350401)
Supplement: S1 Table — (DOCX) [file pone.0350401.s003.docx]

The raw sequencing data generated from this study is available under BioProject ID: PRJNA1287447 and PRJNA1427372 at GenBank (Supplement table S3). (https://www.ncbi.nlm.nih.gov/bioproject/PRJNA1287447)

**Supplement table S3**. Sample metadata and SRA accessions used in this study

| ID | Shelter | BioProject | BioSample | SRA Accession |
| --- | --- | --- | --- | --- |
| A01 | Shelter A | PRJNA1287447 | SAMN49820330 | SRR34394528 |
| A02 | Shelter A | PRJNA1287447 | SAMN49820331 | SRR34394527 |
| A03 | Shelter A | PRJNA1287447 | SAMN49820332 | SRR34394516 |
| A04 | Shelter A | PRJNA1287447 | SAMN49820333 | SRR34394505 |
| A05 | Shelter A | PRJNA1287447 | SAMN49820334 | SRR34394494 |
| A06 | Shelter A | PRJNA1287447 | SAMN49820335 | SRR34394483 |
| A07 | Shelter A | PRJNA1287447 | SAMN49820336 | SRR34394472 |
| A08 | Shelter A | PRJNA1287447 | SAMN49820337 | SRR34394471 |
| A09 | Shelter A | PRJNA1287447 | SAMN49820338 | SRR34394470 |
| A10 | Shelter A | PRJNA1287447 | SAMN49820339 | SRR34394469 |
| A11 | Shelter A | PRJNA1287447 | SAMN49820340 | SRR34394526 |
| A12 | Shelter A | PRJNA1287447 | SAMN49820341 | SRR34394525 |
| A13 | Shelter A | PRJNA1287447 | SAMN49820342 | SRR34394524 |
| A14 | Shelter A | PRJNA1287447 | SAMN49820343 | SRR34394523 |
| A15 | Shelter A | PRJNA1287447 | SAMN49820344 | SRR34394522 |
| A16 | Shelter A | PRJNA1287447 | SAMN49820345 | SRR34394521 |
| A17 | Shelter A | PRJNA1287447 | SAMN49820346 | SRR34394520 |
| A18 | Shelter A | PRJNA1287447 | SAMN49820347 | SRR34394519 |
| A19 | Shelter A | PRJNA1287447 | SAMN49820348 | SRR34394518 |
| A20 | Shelter A | PRJNA1287447 | SAMN49820349 | SRR34394517 |
| B01 | Shelter B | PRJNA1287447 | SAMN49820350 | SRR34394515 |
| B02 | Shelter B | PRJNA1287447 | SAMN49820351 | SRR34394514 |
| B03 | Shelter B | PRJNA1287447 | SAMN49820352 | SRR34394513 |
| B04 | Shelter B | PRJNA1287447 | SAMN49820353 | SRR34394512 |
| B05 | Shelter B | PRJNA1287447 | SAMN49820354 | SRR34394511 |
| B06 | Shelter B | PRJNA1287447 | SAMN49820355 | SRR34394510 |
| B07 | Shelter B | PRJNA1287447 | SAMN49820356 | SRR34394509 |
| B08 | Shelter B | PRJNA1287447 | SAMN49820357 | SRR34394508 |
| B09 | Shelter B | PRJNA1287447 | SAMN49820358 | SRR34394507 |
| B10 | Shelter B | PRJNA1287447 | SAMN49820359 | SRR34394506 |
| B11 | Shelter B | PRJNA1287447 | SAMN49820360 | SRR34394504 |
| B12 | Shelter B | PRJNA1287447 | SAMN49820361 | SRR34394503 |
| B13 | Shelter B | PRJNA1287447 | SAMN49820362 | SRR34394502 |
| B14 | Shelter B | PRJNA1287447 | SAMN49820363 | SRR34394501 |
| B15 | Shelter B | PRJNA1287447 | SAMN49820364 | SRR34394500 |
| B16 | Shelter B | PRJNA1287447 | SAMN49820365 | SRR34394499 |
| B17 | Shelter B | PRJNA1287447 | SAMN49820366 | SRR34394498 |
| B18 | Shelter B | PRJNA1287447 | SAMN49820367 | SRR34394497 |
| B19 | Shelter B | PRJNA1287447 | SAMN49820368 | SRR34394496 |
| B20 | Shelter B | PRJNA1287447 | SAMN49820369 | SRR34394495 |
| C01 | Shelter C | PRJNA1287447 | SAMN49820370 | SRR34394493 |
| C02 | Shelter C | PRJNA1287447 | SAMN49820371 | SRR34394492 |
| C03 | Shelter C | PRJNA1287447 | SAMN49820372 | SRR34394491 |
| C04 | Shelter C | PRJNA1287447 | SAMN49820373 | SRR34394490 |
| C05 | Shelter C | PRJNA1287447 | SAMN49820374 | SRR34394489 |
| C06 | Shelter C | PRJNA1287447 | SAMN49820375 | SRR34394488 |
| C07 | Shelter C | PRJNA1287447 | SAMN49820376 | SRR34394487 |
| C08 | Shelter C | PRJNA1287447 | SAMN49820377 | SRR34394486 |
| C09 | Shelter C | PRJNA1287447 | SAMN49820378 | SRR34394485 |
| C10 | Shelter C | PRJNA1287447 | SAMN49820379 | SRR34394484 |
| C11 | Shelter C | PRJNA1287447 | SAMN49820380 | SRR34394482 |
| C12 | Shelter C | PRJNA1287447 | SAMN49820381 | SRR34394481 |
| C13 | Shelter C | PRJNA1287447 | SAMN49820382 | SRR34394480 |
| C14 | Shelter C | PRJNA1287447 | SAMN49820383 | SRR34394479 |
| C15 | Shelter C | PRJNA1287447 | SAMN49820384 | SRR34394478 |
| C16 | Shelter C | PRJNA1287447 | SAMN49820385 | SRR34394477 |
| C17 | Shelter C | PRJNA1287447 | SAMN49820386 | SRR34394476 |
| C18 | Shelter C | PRJNA1287447 | SAMN49820387 | SRR34394475 |
| C19 | Shelter C | PRJNA1287447 | SAMN49820388 | SRR34394474 |
| C20 | Shelter C | PRJNA1287447 | SAMN49820389 | SRR34394473 |
